# Synthesis, Antimicrobial Screening, Homology Modeling, and Molecular Docking Studies of a New Series of Schiff Base Derivatives as Prospective Fungal Inhibitor Candidates

**DOI:** 10.3390/molecules24183250

**Published:** 2019-09-06

**Authors:** Yahya Toubi, Farid Abrigach, Smaail Radi, Faiza Souna, Abdelkader Hakkou, Abdulrhman Alsayari, Abdullatif Bin Muhsinah, Yahia N. Mabkhot

**Affiliations:** 1 Laboratory of Applied Chemistry & Environment, Faculty of Sciences, Mohammed First University, Oujda 60000, Morocco; 2 Laboratory of Biochemistry, Faculty of Sciences, Mohammed First University, Oujda 60000, Morocco (F.S.) (A.H.); 3 Department of Pharmacognosy, College of Pharmacy, King Khalid University, Abha 61441, Saudi Arabia (A.A.) (A.B.M.); 4 Department of Pharmaceutical Chemistry, College of Pharmacy, King Khalid University, Abha 61441, Saudi Arabia

**Keywords:** Schiff bases, synthesis, antifungal, molecular docking, homology modeling, pharmacophore site

## Abstract

Twelve new Schiff base derivatives have been prepared by the condensation reaction of different amino substituted compounds (aniline, pyridin-2-amine, *o*-toluidine, 2-nitrobenzenamine, 4-aminophenol, and 3-aminopropanol) and substituted aldehydes such as nicotinaldehyde, *o*,*m*,*p*-nitrobenzaldehyde, and picolinaldehyde in ethanol using acetic acid as a catalyst. The envisaged structures of the all the synthesized ligands have been confirmed on the basis of their spectral analysis FT-IR, mass spectroscopy, ^1^H- and ^13^C-NMR. In vitro screening of their antibacterial and antifungal potential against *Escherichia coli* bacterium and *Fusarium oxysporum f.sp albedinis* (*F.o.a*) fungus, respectively, revealed that all the ligands showed no significant antibacterial activity, whereas most of them displayed good antifungal activity. Homology modeling and docking analysis were performed to explain the antifungal effect of the most and least active compound against two *F.o.a* fungus proteins.

## 1. Introduction

Nowadays, much attention is paid to the study of Schiff base compounds, not only for their broad range of biological activities—such as antimalarial, antiviral, antitubercular, anticancer, anti-inflammatory, antifungal, and antibacterial properties [1,2,3,4,5,6]—but also because they are widely used as catalyst carriers, optical chemical sensors, thermo-stable materials, metal complexion agents, corrosion inhibitors, and polymer stabilizers [7,8,9,10]. Such Schiff bases, also known as imines or azomethines, can be obtained easily via the condensation reaction between primary amines and carbonyl compounds. In addition, some works reveal that several natural compounds containing azomethine groups form compounds of high pharmacological interest [11,12]. Their critical biological activities are due principally to the presence of the imine group (C=N) [13].

In the case of infections, the search for potent and selective inhibitors has received particular attention because of the increase of antimicrobial resistance, which has become one of the biggest challenges to global health, food security, and development. In particular, and concerning the agricultural area, *Fusarium oxysporum f.sp albedinis (F.o.a)* represents the leading dangerous agent among all pathologies of date palms cultivation, particularly in North Africa [14]. In the last century, Bayoud disease, which is caused by the telluric fungus *F.o.a*, has destroyed more than 15 million Moroccan and Algerian date palm trees. Unfortunately, to this day, no curative treatment yet exists against this fungus, except some limited methods such as disinfection of the soil, propagation. and use of resistant varieties—which remains the first way to reduce the impact of this disease [15].

On the other hand, due to incomplete information on the pathogenesis of the *F.o.a* fungus, various computational techniques can be used in order to better understand the mechanism of action of this fungus. In this context, molecular docking simulation remains one of the most powerful tools that can give an atomistic insight into molecular recognition by predicting the ability of a molecule to bind to the active site of a protein. In our case, two *F.o.a* proteins were chosen as targets for our docking studies. The first one was the Fusarium oxysporum Guanine nucleotide-binding protein (Fgb1), a membrane protein of high importance in many biological processes of *F. oxysporum* fungal. The disruption of this protein leads to pathogenicity reduction and alterations in physiological characteristics, like heat resistance and germination frequency [16]. The second target was the Fusarium oxysporum phytase domain (Fophy) enzyme, another protein that plays versatile roles in agricultural and feeding fields. It catalyzes the degradation of phytate (an important constituent of grains, cereals, and oilseeds) into inorganic phosphorus and myoinositol phosphate derivatives. Inhibition of the Fophy enzyme can affect the growth of the fungus indirectly by stopping the degradation of phytate, which is well-known as a strong chelating agent and readily binds covalent metal ions, rendering them insoluble and thus unavailable for absorption [17,18]. In general, these facts make both of these proteins as prospective potential targets to develop new anti-*F.oxysporum* inhibitors.

Accordingly, and keeping in mind the biological significance of the Schiff base derivatives, we report in this paper the synthesis of new imines compounds as potent antimicrobial agents. In addition, a molecular docking approach was performed for the most and the least antifungal derivatives in the homology models we constructed for Fusarium oxysporum Guanine nucleotide-binding protein (Fgb1) and Fusarium oxysporum phytase domain (Fophy), to gain better insight on the ligand–receptor binding interactions and also to direct future syntheses.

## 2. Results and Discussion

### 2.1. Chemistry

**Scheme 1:**
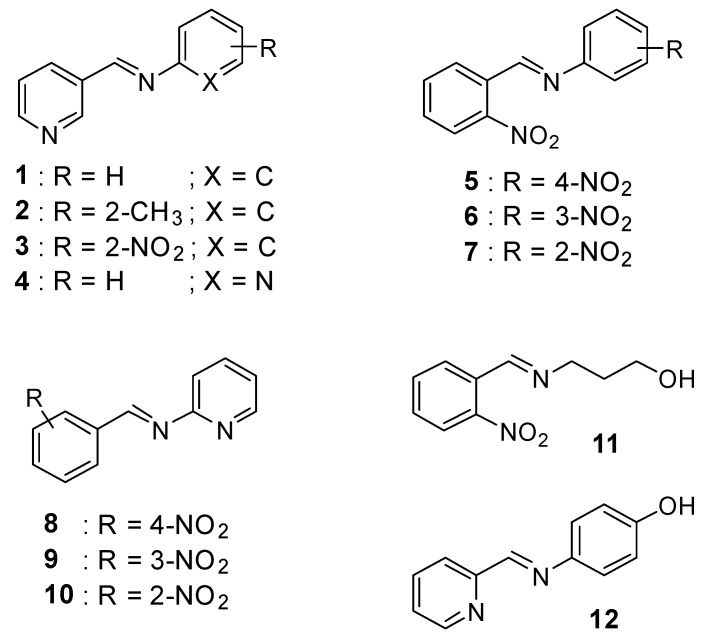
General structures of the prepared Schiff base derivatives.

The new Schiff bases **1**–**12** were synthesized by condensation reaction of primary amines (aniline, pyridin-2-amine, o-toluidine,2-nitrobenzenamine, 4-aminophenol, and 3-aminopropan-1-ol) and substituted aldehydes (nicotinaldehyde, *o*,*m*,*p*-nitrobenzaldehyde, and picolinaldehyde) in ethanol using acetic acid as a catalyst to obtain the desired compounds in nearly 79–96% yield. The ligands were quite stable at room temperature in the solid state. The spectroscopic data are in total agreement with the proposed structures, as shown in Scheme 1.

The NMR spectra of all compounds were recorded on a Bruker AC 300 spectrometer. TMS was used as the reference standard and the solvent was CDCl_3_. The ^1^H-NMR spectra clearly indicate the presence of the characteristic peak of the alpha–hydrogen of CH=N as a singlet, which remained a strong indication of the formation of the desired Schiff base derivatives **1**–**12**. The chemical shift values of these alpha–hydrogen were found to be ranged from 7.37 ppm for compound **1** to 8.75 ppm for compound **6**. In general, the observed differences in the values of chemical shifts of the signal under study can be explained by the shielding and deshielding effect of the CH=N proton, probably due to the different electronic effects introduced by the substituent groups on the phenyl rings of each compound. In fact, the presence of one or more electron withdrawing groups such as NO_2_ (compounds **5**–**12**) leads to a decrease of the electron density on the C=N proton, which makes it more deshielded than the proton in the case of compounds **1** and **2,** which showed chemical shifts of 7.37 and 7.45 ppm, respectively, which are less than that observed in the case of compounds **5**–**12** (up to 8 ppm).

### 2.2. Biological Activity

The antimicrobial potentials of the titled compounds were checked against the *E.coli* bacterium strain and the *Fusarium oxysporum f.sp albedinis* fungus, utilizing the agar diffusion method.

Unfortunately, the results of the preliminary screening showed that none of the tested compounds were found to exhibit antibacterial effect against the used bacterium. These results can be explained by the high resistance of the *E.coli* strain to this kind of product, or also by the absence of active or pharmacophore sites which can act as specific and potential features to inhibit the growth of this bacterium [19].

On the contrary, the results of the in vitro antifungal assay revealed that most of the screened ligands showed high to moderate activities against *F.o.a* based on their minimum inhibitory concentration (MIC) values. The results obtained from the evaluation study are provided in Table 1. The maximum activity was 0.02 µg·mL^−1^, shown by compound **3**, followed by compounds **6**, **7,** and **12** with MIC values equal to 0.04 µg·mL^−1^, while compound **2** showed the least MIC value (i.e., 0.9 µg·mL^−1^). Other products also have considerable activities, with MICs varying from 0.08 µg·mL^−1^ for compound **11** to 0.30 µg·mL^−1^ for compound **5**. Comparing both the structures of **2** and **3,** we can conclude that the presence of a strong electron withdrawing group like nitro moiety (NO_2_) at the ortho position of the phenyl ring is very suitable to increase the antifungal efficiency; in time the presence of an electron donating group such as methyl moiety (CH_3_) is unfavorable for antifungal activity. However, unfortunately, with these preliminary investigations, it is difficult to determine the right factors that affect the antifungal potential of these derivatives. For this, more investigations are required using other models and methods.

### 2.3. Computational Studies

#### 2.3.1. Molecular Electrostatic Potential (MEP) Maps

**Figure 1:**
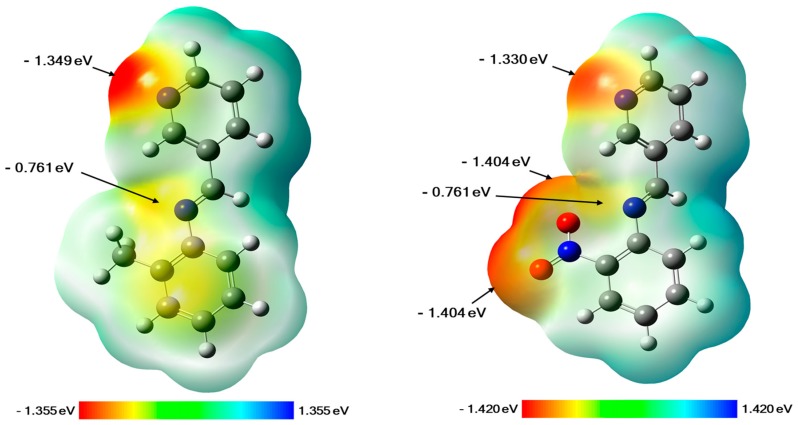
Generated molecular electrostatic potential (MEP) maps of compound **2** (**left**) and compound **3** (**right**). The negative (red, orange, and yellow) regions are related to electrophilic reactivity, whereas positive (green and blue) regions accompany nucleophilic reactivity.

Molecular electrostatic potential (MEP) is a very powerful tool utilized to determine or predict the reactive regions of nucleophilic and electrophilic attacks on a molecular system. It can be generated by mapping the electrostatic potential on to the isoelectron density surface of the molecule, which gives us the possibility to determine the distribution of the electronic charge over all the structure. Actually, MEP mapping remains a useful way to understand the interaction of a molecule with its environment and to study the hydrogen bonding interactions, as well as the biological recognition processes [20]. In our case, the MEP maps of both compounds **2** and **3** were generated based on their DFT (Density Functional Theory) optimized geometries, and they are displayed in Figure 1. As can be seen for compound **2**, the negative electrostatic potential regions are mainly concentered over the nitrogen atoms of the pyridine ring and the imine function with values of −1.349 and −0.761 eV (at an isovalue of 0.0004 electrons/Å^3^), respectively. While for compound **3**, the negative charges are located on the nitrogen atoms of the pyridine ring and the imine function, as well as the oxygen atoms of the nitro group (NO_2_) at the ortho position of the phenyl ring, with values ranging from −1.404 to −0.741 eV. These results give us valuable information about the possible sites that can be involved in interactions by hydrogen bonds with the amino acid residues of protein receptors.

#### 2.3.2. Homology Modeling

**Figure 2:**
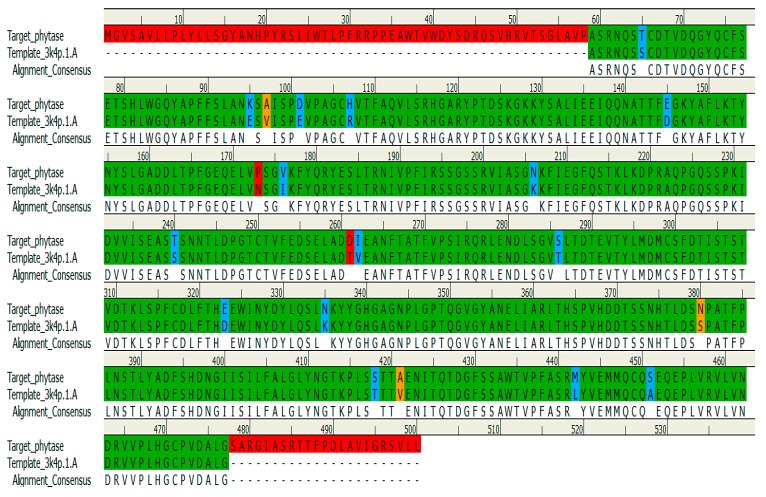
The alignment of Fusarium oxysporum phytase domain and Aspergillus Niger Phytase template (PDB code: 3K4P) that yielded the homology model of Fophy. The identical residues are represented by green color, residues with strong similarity are represented by blue, and residues with weak identity are in orange, whereas the red color represents the non-matching residues.

In order to gain better insights and to better understand the binding mode of the title compounds **2** and **3** towards both the Fusarium oxysporum Guanine nucleotide-binding protein beta subunit (Fgb1) and the Fusarium oxysporum isolate FO 3-phytase-like (Fophy) proteins, it was planned to perform molecular docking simulations. However, because of the unavailability of experimental crystal structures of these proteins, homology modeling studies were carried out to construct three-dimensional (3D) structures of Fgb1 and Fophy prior to use in our docking studies. For Fgb1 protein, the model was generated and validated as described previously in our last works [21,22] using the crystal structure of the Rattus norvegicus Guanine nucleotide-binding protein G(I)/G(S)/G(T) subunit beta-1 (3SN6_chain B) as a suitable template. Following the same procedure described for the Fgb1 model, the 3D structure of the Fophy protein was also built. In brief, the DNA sequence of Fophy was selected as a target and it was downloaded from the NCBI GenBank database with accession number KC708486, and then converted to its equivalent amino acid sequence before running a BLASTp search. Aspergillus Niger Phytase (3K4P_chain A) was found the best-matched template with a 95.2% similarity to the Fophy sequence and with a resolution of 2.4 Å, making it an excellent template. The target and the template protein sequences were then aligned with the aid of the Clustal Omega and visualized by DS programs (Figure 2).The resulting sequence alignment shows a total conservation of 79.8% (399 residues), strong similarity of 3% (15 residues), and weak identity of 0.6% (3 residues) of amino acid between the Fophy sequence and that of the 3K4P_chain A—whilst 83 amino acids (16.6%) of Fophy do not match those found in the selected template sequence.

**Figure 3:**
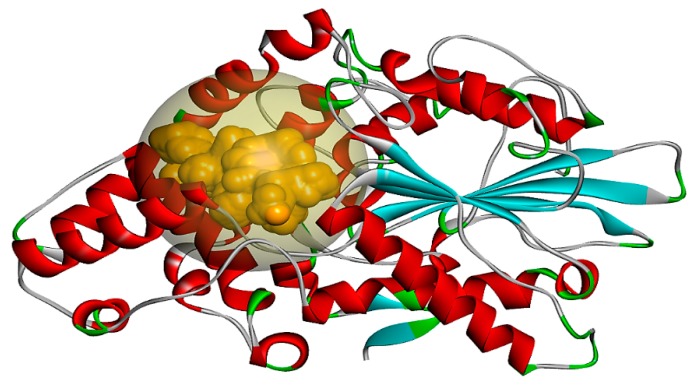
Modeled structure of the Fophy protein based on the aligned sequence between Fophy and Aspergillus Niger Phytase (3K4P_chain A) sequences.

Figure 3 shows the modeled 3D structure of Fophy based on the aligned sequence; the red color indicates α-helices and the light blue color indicates β-sheets, whereas β-turns are indicated by green color. All these elements are connected by loops (in light gray color). All α- helices, β-sheets, β-turns, and random coils (loops) match the same alignment found in the template structure. The binding site is shown as an orange transparent sphere.

**Figure 4:**
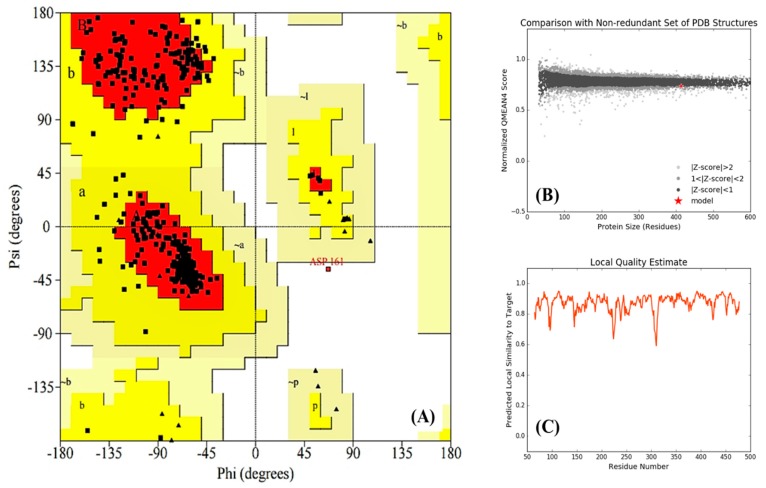
Validation of the generated homology model of Fophy. (**A**) Ramachandran plot, (**B**) comparison plot, and (**C**) local quality plot. For more information about Ramachandran plot descriptions the reader should be referred to the web site at (https://www.ebi.ac.uk/thornton-srv/databases/cgi-bin/pdbsum/GetPage.pl?doc=TRUE&pdbcode=n/a&template=doc_procheck01.html).

Validation of the final model was performed by analyzing its Ramachandran plot (Figure 4A), comparison plot (Figure 4B), and local quality plot (Figure 4C). The resulting Ramachandran plot shows that more than 90% of amino acid residues are distributed in the most favored regions (red color) and 9.4% of residues are in additional allowed regions (yellow color)—while none or few residues are located in generously allowed regions (light yellow color) and disallowed regions (white color), respectively. In the comparison plot, every dot (black, gray, or light gray) represents one experimental protein structure, where the analyzed model is represented as a red star. From this plot, it can be concluded that our model is comparable to the experimental protein structures because the red star is on the experimental line. In addition, the local quality plot shows, for each residue of the generated model, the predictable similarity to the native structure. Typically, residues showing a score up to 0.6 are expected to be of good quality. In our case, as shown in Figure 4C, all residues indicate similarities to the target above 0.6, which indicates that the generated model is in the norms of good structures.

Finally, by considering all these results of the Ramachandran, comparison, and local quality plots, we can suggest that the built model is reasonably good in geometry and stereochemistry, and is very appropriate to examine the next step, which is the ligand–protein docking studies of our compounds.

#### 2.3.3. Molecular Modeling Study

Molecular docking calculations were performed to identify selective compounds that could be interacting with the catalytic sites of the Fgb1 and the Fophy proteins. In this study, flexibility was allowed in all the rotable bonds of the ligand, while the protein was used as a rigid structure. 

##### Docking against the Fgb1 Protein

**Figure 5:**
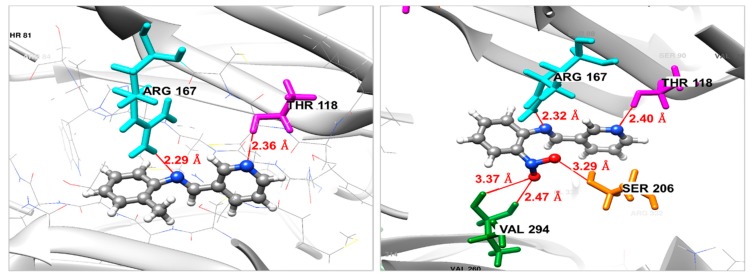
Binding mode of compound **2** (**left**) and compound **3** (**right**) in the active site of Fgb1 protein. For both **2** and **3**, gray color for carbon atoms, blue for nitrogen, red for oxygen and write color for hydrogen atoms.

The binding modes of the selected molecules (compounds **3** and **2**) in the protein active pocket of the modeled Fgb1 are depicted in Figure 5. The binding energy ΔG_binding_ has been used to evaluate the binding affinity of each molecule to the Fgb1 protein. From the docking study, it was found that the ligand **3** shows the best affinity towards the Fgb1 protein with a ΔG_binding_ = −7.34 kcal/mol, when compared to the compound **2**, which shows a ΔG_binding_ of −6.94 kcal/mol. As can be observed from Figure 5, compound **3** forms five H-bond interactions with the amino acid residues of the receptor—one between the nitrogen atom of the imine function and the hydrogen atom of Arg167 with a distance of 2.32 Å, and with a distance of 2.40 Å, the nitrogen atom of the pyridine ring made the second H-bond with amino acid Thr118. Moreover, the pharmacophoric nitro group at the ortho position of the phenyl ring interacts via three H-bonds, two with the residue Val294 (2.47 and 3.37 Å) and one with Ser206 residue with a distance of 3.29 Å. In contrast, compound **2** forms only two H-bond interactions between the residues Arg167 and Thr118 and nitrogen atom of the imine function and the pyridine ring, respectively. From these findings, we can see that the ligand–protein complex is stabilized mainly by hydrogen bonds, especially those that the nitro group forms with Val294 and Ser206, which mean that this nitro moiety helps to maintain the molecule in the binding pocket and makes it more active against the studied target.

##### Docking Against the Fophy Protein

As second target protein, Fusarium oxysporum isolate FO 3-phytase-like, was chosen to perform a docking study of the most and the least active compounds, as mentioned previously. The parameters that determine the binding affinity of each compound to the active pocket of the Fophy protein were calculated and are reported in the Table 2.

**Figure 6:**
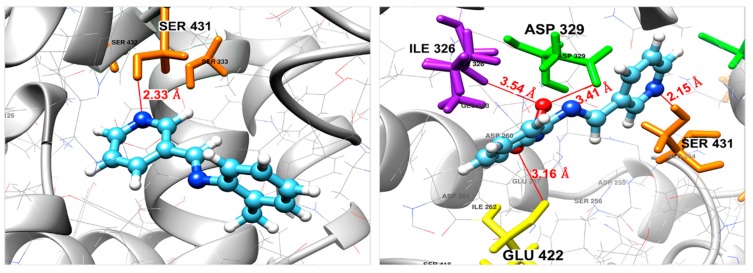
Binding mode of compound **2** (**left**) and compound **3** (**right**) in the active site of the Fophy protein. For both **2** and **3**, light blue color for carbon atoms, blue for nitrogen, red for oxygen and write color for hydrogen atoms.

The most active compounds, i.e., **3,** gave a calculated docking score (ΔG_binding_) of −6.78 kcal/mol, higher than that shown by compound **2,** which is equal to −5.84 kcal/mol. This finding indicates that compound **3**, in its optimal docked pose, is more attached (more active) to the Fophy protein than compound **2**, which is in total agreement with our experimental results. The optimal binding of **3** appears to arise from four principal interactions, specifically, one of the nitro group oxygen interacts with Glu422 of the Fophy active domain via the formation of a medium–strong halogen bonding interaction (3.16 Å). The other oxygen of the nitro group forms a halogen bond with Asp329 (3.41 Å), as well as a hydrogen bond with Ile326 (3.54 Å). The interactions are also stabilized by another H-bond formed between the nitrogen atom of the pyridine ring and the hydrogen atom of Ser431 residue with a distance of 2.15 Å. On the other hand, compound **2** was found to be attached to the active pocket of Fophy with a single strong H-bond between the nitrogen atom of the pyridine ring and Ser431 residue with a distance of 2.15 Å. The 3D binding models of both compounds **2** and **3** in the Fophy homology model are shown in Figure 6.

For comparison, the disappearance or the absence of interactions between the nitrogen atom of the imine function and the active site residues in the case of the Fophy protein, in comparison with the case of the Fgb1 protein, can be explained by the absence of amino acid residues close to this atom, which makes the formation of such interactions impossible or more difficult. Also, it is important to mention that all the interactions formed between the ligands and the amino acids of the binding pocket of both studied proteins occurred by the ligand’s atoms with high electron densities, as mentioned by the generated MEP maps.

Finally, in general, all these important findings and theoretical observations open up the way for more chemical investigations, in order to make our compounds even more efficient, and also present a guideline to facilitate a better understanding of the mode of action of the ligand−enzyme complex and give a clear insight into the design of new molecules in the future. Furthermore, within the general context of development of new anti-Fusarium agents targeting proteins and specially Fgb1, additional suitable experimental assays are required to confirm the good capacity of these active compounds.

## 3. Materials and Methods

### 3.1. General Information

All solvents and other chemicals, obtained from usual commercial sources, were of analytical grades and used without further purification. Melting points were determined by using a BUCHÏ 510 m.p. apparatus (FSO, Oujda, Morocco). The NMR spectra were obtained with a Bruker AC 300 spectrometer (CNRST, Rabat, Morocco). Infrared (FT-IR) spectra were acquired on a Shimadzu infrared spectrophotometer (FSO, Oujda, Morocco) using the KBr disc technique. Molecular weights were determined by a JEOL JMS DX-300 Mass Spectrometer (CNRST, Rabat, Morocco).

### 3.2. General Procedure for the Synthesis of Compounds ***1***–***12***

An equal molar solution of a primary amine (0.01 mol) and an aldehyde (0.01 mol) in dry methanol (50 mL) was refluxed for 3 h using a few drops of acetic acid as a catalyst. The reaction mixture was then cooled to room temperature and the formed precipitate was filtered, washed with methanol (35 mL) and diethyl ether (2 × 5 mL), and then dried under vacuum. The recrystallization from methanol/dioxane (1:4) mixture gave pure products (**1**–**12**).

*N*-((*E*)-pyridin-3-ylmethylidene)aniline (**1**): White Powder. Yield 80%; m.p. 123–126 °C; IR (KBr, cm^−1^): 3082 (C-H, arom); 1602 (C=N, arom); 1597 (C=N); 1570 (C=C); ^1^H-NMR (300 MHz, CDCl_3_, ppm): 7.3 (m, 1H, benzene), 7.37 (s, CH=N, imine), 8.8 (m, 1H, pyridine); ^13^C-NMR (75 MHz, CDCl_3_, ppm): 122.1 (C14-benzene), 122.3 (C10-benzene), 123.9 (C5-pyridine), 127.3 (C12-benzene), 130.1 (C13, C11-benzene), 130.2 (C3-pyridine), 130.4 (C4-pyridine), 137.2 (C9-benzene), 149 (C2-pyridine), 151.5 (C6-pyridine), 152 (C7-imine). Mass spectrum (EIMS) [M]^+^ = 183.

2-methyl-*N*-((*E*)-pyridin-3-ylmethylidene)aniline (**2**): White powder. Yield 92%; m.p. 176–181 °C; IR (KBr, cm^−1^): 3080 (C-H, arom); 2965(C-H, CH_3_); 1609 (C=N, arom); 1592 (C=N); 1568 (C=C); ^1^H-NMR (300 MHz, CDCl_3_, ppm): 7.4 (m, 1H, methylbenzene), 7.45 (s, CH=N, imine), 8.8 (m, 1H, pyridine), 2.4 (s, 3H, CH_3_); ^13^C-NMR (75 MHz, CDCl_3_, ppm): 15.7 (C15- methylbenzene), 122.2 (C14- methylbenzene), 123.9 (C5-pyridine), 127.1 (C13-methylbenzene), 127.2 (C12-methylbenzene), 130.4 (C11-benzene, C3-pyridine), 130.6 (C10-methylbenzene), 137.2 (C4-pyridine), 147 (C9- methylbenzene), 151.5 (C2-pyridine), 152 (C6-pyridine), 155.92 (C7-imine). Mass spectrum (EIMS) [M]^+^ = 167.

2-nitro-*N*-((*E*)-pyridin-3-ylmethylidene)aniline (**3**): Crystal. Yield 85%; m.p. 145–150 °C; IR (KBr, cm^−1^): 3080 (C-H, arom); 1609 (νC=N, arom); 1592 (C=N); 1568 (C=C); 1360 (C-NO_2_, arom); ^1^H-NMR (300 MHz, CDCl_3_, ppm): 6.8 (m, 1H, nitrobenzene), 7.48 (s, CH=N, imine), 8.8 (m, 1H, pyridine); ^13^C-NMR (75 MHz, CDCl_3_, ppm): 122.4 (C11-nitrobenzene), 123.2 (C14-nitrobenzene), 123.9 (C12-nitrobenzene), 128.2 (C3-pyridine), 130.4 (C13-nitrobenzene), 136.2 (C4-pyridine), 137.2 (C10-nitrobenzene), 141.9 (C9-nitrobenzene), 144.1 (C2-pyridine), 151.5 (C6-pyridine), 154.69 (C7-imine). Mass spectrum (EIMS) [M]^+^ = 228.

*N*-((*E*)-pyridin-3-ylmethylidene)pyridin-2-amine (**4**): White powder. Yield 96%; m.p. 119–125 °C; IR (KBr, cm^−1^): 3084 (C-H, arom); 1610 (C=N, arom); 1597 (C=N); 1570 (C=C); ^1^H-NMR (300 MHz, CDCl_3_, ppm): 7.8 (m, 1H, pyridine), 8.50 (s, CH=N, imine), 8.8 (m, 1H, pyridine); ^13^C-NMR (75 MHz, CDCl_3_, ppm): 116.2 (C3-pyridine), 122.4 (C5-pyridine), 123.9 (C13-pyridine), 130.4 (C9-pyridine), 137.2 (C14-pyridine), 137.3 (C4-pyridine), 150.4 (C6-pyridine), 151.5 (C10-pyridine), 152 (C12-pyridine), 156.5 (C2-pyridine), 160 (C7-imine). Mass spectrum (EIMS) [M]^+^ = 184.

2-nitro-*N*-((*E*)-(4-nitrophenyl)methylidene)aniline (**5**): Yellow powder. Yield 85%; m.p. 145–150 °C; IR (KBr, cm^−1^): 3080 (C-H, arom); 1609 (C=N, arom); 1592 (C=N); 1568 (C=C); 1360 (C=NO_2_, arom); ^1^H-NMR (300 MHz, CDCl_3_, ppm): 8.2 (m, 1H, nitrobenzene), 8.10 (s, CH=N, imine); ^13^C-NMR (75 MHz, CDCl_3_, ppm): 121.1 (C13, nitrobenzene), 121.2 (C11, nitrobenzene), 122.4 (C3, nitrobenzene), 123.2 (C6, nitrobenzene), 128.2 (C4, nitrobenzene), 130.1 (C14, nitrobenzene), 130.2 (C10, nitrobenzene), 136.2 (C5, nitrobenzene), 139.9 (C9, nitrobenzene), 141.9 (C2, nitrobenzene), 148.3 (C1, nitrobenzene), 150.35 (C8, imine), 150.7(C12, nitrobenzene). Mass spectrum (EIMS) [M]^+^ = 272.

2-nitro-*N*-((*E*)-(3-nitrophenyl)methylidene)aniline (**6**): Yellow powder. Yield 96%; m.p. 181–183 °C; IR (KBr, cm^−1^): 3080 (C-H, arom); 1609 (C=N, arom); 1592 (C=N); 1568 (C=C); 1360 (C=NO_2_, arom); ^1^H-NMR (300 MHz, CDCl_3_, ppm): 8.2 (m, 1H, nitrobenzene), 8.75 (s, CH=N, imine); ^13^C-NMR (75 MHz, CDCl_3_, ppm): 122.4 (C13, nitrobenzene), 123.2 (C11, nitrobenzene), 123.4 (C3, nitrobenzene), 124.1 (C6, nitrobenzene), 128.2 (C4, nitrobenzene), 129.8 (C14, nitrobenzene), 134.7 (C10, nitrobenzene), 135.3 (C5, nitrobenzene), 136.2 (C9, nitrobenzene), 141.8 (C2, nitrobenzene), 148.3 (C1, nitrobenzene), 148.42 (C8, imine), 148.5(C12, nitrobenzene). Mass spectrum (EIMS) [M]^+^ = 272.

2-nitro-*N*-((*E*)-(2-nitrophenyl)methylidene)aniline (**7**): Yellow powder. Yield 83%; m.p. 181–183 °C; IR (KBr, cm^−1^): 3080 (C-H, arom); 1609 (C=N, arom); 1592 (C=N); 1568 (νC=C); 1360 (C-NO_2_, arom); ^1^H-NMR (300 MHz, CDCl_3_, ppm): 8.2 (m, 1H, nitrobenzene), 8.05 (s, CH=N, imine); ^13^C-NMR (75 MHz, CDCl_3_, ppm): 122.4 (C13, nitrobenzene), 123.2 (C11, nitrobenzene), 123.4 (C3, nitrobenzene), 124.1 (C6, nitrobenzene), 128.2 (C4, nitrobenzene), 129.8 (C14, nitrobenzene), 134.7 (C10, nitrobenzene), 135.3 (C5, nitrobenzene), 136.2 (C9, nitrobenzene), 141.8 (C2, nitrobenzene), 148.3 (C1, nitrobenzene), 145 (C8, imine), 148.5(C12, nitrobenzene). Mass spectrum (EIMS) [M]^+^ = 272.

*N*-((*E*)-(4-nitrophenyl)methylidene)pyridin-2-amine (**8**): Crystal. Yield 85%; m.p. 150–155 °C; IR (KBr, ν cm^−1^): 3084 (C-H, arom); 1601 (C=N, arom); 1590 (C=N); 1562 (νC=C); 1364 (νC=NO_2_, arom); ^1^H-NMR (300 MHz, CDCl_3_, ppm): 8.2 (m, 1H, nitrobenzene), 8.10 (s, CH=N, imine), 7.8 (m, 1H, pyridine); ^13^C-NMR (75 MHz, CDCl_3_, ppm): 116.2 (C11-C13, nitrobenzene), 121.2 (C10, nitrobenzene), 121.3 (C14, nitrobenzene), 130.1 (C2, pyridine), 137.3 (C3-C5, pyridine), 139.9 (C4, pyridine), 148.42 (C8, imine), 150.4 (C6, pyridine),150.7 (C9, nitrobenzene), 160.7 (C12, nitrobenzene). Mass spectrum (EIMS) [M]^+^ = 228.

*N*-((*E*)-(3-nitrophenyl)methylidene)pyridin-2-amine (**9**): Crystal. Yield 79%; m.p. 142–145 °C; IR (KBr, ν cm^−1^): 3085 (C-H, arom); 1606 (C=N, arom); 1597 (C=N); 1565 (C=C); 1364 (C-NO_2_, arom); ^1^H-NMR (300 MHz, CDCl_3_, ppm): 8.5 (m, 1H, nitrobenzene), 8.15 (s, CH=N, imine), 8.00 (m, 1H, pyridine); ^13^C-NMR (75 MHz, CDCl_3_, ppm): 116.2 (C11-C13, nitrobenzene), 121.2 (C10, nitrobenzene), 121.3 (C14, nitrobenzene), 130.1 (C2, pyridine), 137.3 (C3-C5, pyridine), 139.9 (C4, pyridine), 148.42 (C8, imine), 150.4 (C6, pyridine),150.7 (C9, nitrobenzene), 160.7 (C12, nitrobenzene). Mass spectrum (EIMS) [M]^+^ = 228.

*N*-((*E*)-(2-nitrophenyl)methylidene)pyridin-2-amine (**10**): Yellow powder. Yield 95%; m.p. 208–212 °C; IR (KBr, ν cm^−1^): 3085 (C-H, arom); 1606 (C=N, arom); 1597 (C=N); 1565 (C=C); 1364 (C=NO_2_, arom); ^1^H-NMR (300 MHz, CDCl_3_, ppm): 8.2 (m, 1H, nitrobenzene), 8.6 (s, CH=N, imine), 8.05 (m, 1H, pyridine); ^13^C-NMR (75 MHz, CDCl_3_, ppm): 116.2 (C11-C13, nitrobenzene), 121.2 (C10, nitrobenzene), 121.3 (C14, nitrobenzene), 130.1 (C2, pyridine), 137.3 (C3-C5, pyridine), 139.9 (C4, pyridine), 148.42 (C8, imine), 150.4 (C6, pyridine),150.7 (C9, nitrobenzene), 160.7 (C12, nitrobenzene). Mass spectrum (EIMS) [M]^+^ = 228.

3-(((*Z*)-(2-nitrophenyl)methylidene)amino)propan-1-ol (**11**): Yellow powder. Yield 85%; m.p. 187–190 °C; IR (KBr, ν cm^−1^): 3500 (O-H); 3078 (C-H, arom); 2870 (C-H_2_); 1597 (C=N); 1565 (C=C); 1364 (C-NO_2_, arom); 1070 (C-O); ^1^H-NMR (300 MHz, CDCl_3_, ppm): 7.8 (m, 1H, nitrobenzene), 8.7 (s, CH=N, imine), 3.55(N-CH_2_-CH_2_-CH_2_-O), 1.84 (N-CH_2_-CH_2_-CH_2_-O), 3.53(N-CH_2_-CH_2_-CH_2_-O), 2.1 (s, OH); ^13^C-NMR (75 MHz, CDCl_3_, ppm): 34.2 (C12, propanol), 57.9 (C11, propanol), 60.5(C13, propanol), 122.4(C6, nitrobenzene), 123.2 (C3, nitrobenzene), 128.2 (C5, nitrobenzene), 136.2 (C2, nitrobenzene), 141.9 (C4, nitrobenzene), 148.3 (C1, nitrobenzene), 157.20 (C9, nitrobenzene). Mass spectrum (EIMS) [M]^+^ = 209.

4-(((*E*)-pyridin-2-ylmethylidene)amino)phenol (**12**): White powder. Yield 80%; m.p. 185–188 °C; IR (KBr, ν cm^−1^): 3550 (O-H); 3068 (C-H, arom); 1587 (C=N); 1568 (C=C); 1072 (C-O); ^1^H-NMR (300 MHz, DMSO, ppm): 7.8 (m, 1H, phenol), 7.8 (m, 1H, pyridine), 8.13 (s, CH=N, imine), ^13^C-NMR (75 MHz, CDCl_3_, ppm): 117.3 (C11-C13, phenol), 123.8 (C10-C14, phenol), 123.9 (C3, pyridine), 126.2 (C5, pyridine), 136.1 (C4, pyridine), 141.6 (C9, phenol), 149.2 (C6, pyridine), 137.83 (C7, imine), 153.6 (C2, pyridine), 157 (C12, phenol). Mass spectrum (EIMS) [M]^+^ = 199.

### 3.3. Biological Evaluation

#### 3.3.1. In Vitro Antifungal Assay

The in vitro antifungal activities of all the new compounds were determined by the agar diffusion technique against *Fusarium oxysporum f.sp albedinis* (*F.o.a*) fungus, as previously described [23,24]. For tests, the agar media (potato dextrose agar) were inoculated with a solution of the tested compound in DMSO/EtOH (50/50) at different concentrations, and then mycelial discs of 6 mm diameter of the *F.o.a* fungus were placed into the middle of each Petri plate. Thereafter, all the plates were incubated at 28 °C for 7 days. The growth of the microorganism was followed by a measurement of mycelium diameter. The inhibition percentage of a molecule is equal to the ratio of the mycelium diameter of the culture in the presence of a dose of the tested compound over the mycelium diameter of the reference culture multiplied by 100. The minimum inhibition concentration (MIC) is the least dose of the compound which caused inhibition of the microorganism growth.

$$\mathrm{Inhibition}\mathrm{percentage}\;=\;(\frac{\mathrm{Do}-\mathrm{Dx}}{\mathrm{Do}})\times 100$$

where Do and Dx are the diameters of the mycelial growth of the *F.o.a* in the absence and in the presence of the tested compound, respectively. Due to lack of a known antibiotic that can treat this infection directly, (DMSO/EtOH + distilled water) mixture was used as negative control without any standard reference drug.

#### 3.3.2. Antibacterial Test

The prepared compounds were screened for their in vitro antibacterial potential against *Escherichia coli* bacterium strain in accordance with the National Committee for Clinical Laboratory Standards (NCCLS) recommendations [25] using the disc diffusion method. Briefly, overnight culture of *E.coli* strain was subcultured on Muller–Hinton agar into Petri plates. Sterile WHATMAN paper discs of 6 mm diameter were put in the middle of each plate and then soaked up by DMSO/EtOH solution of the tested compounds at different concentrations. The incubation was carried out at 37 °C for 24 h. The zones of inhibition around each disc were noted.

### 3.4. Computational Studies

#### 3.4.1. Ligand’s Preparation

The structures of all Schiff base derivatives were drawn using ACD/ChemSketch software (version 12.01(free version), FSO, Oujda, Morocco) [26]. Geometry optimization was done with the aid of the Gaussian 09 package (FSO, Oujda, Morocco) [27] at DFT/B3LYP level with 6-31G(d,p) basis. GaussView 06 software was used to calculate and generate the molecular electrostatic potential (MEP) maps of the optimized structures.

#### 3.4.2. Homology Modeling

Both the Fusarium oxysporum Guanine nucleotide-binding protein beta subunit (Fgb1) and the Fusarium oxysporum isolate FO 3-phytase-like (Fophy) proteins were used in the present work. The target primary amino acid sequence of Fgb1 was retrieved from the UniProtKB database (accession number Q96VA6), while the DNA sequence of Fophy was received from the NCBI GenBank database with accession number KC708486, and then translated to the equivalent amino acid sequence with the aid of Discovery Studio client v16 software (DS) (version 16 (academic version), Oujda, Morocco) [28]. To find suitable homologues sequences (templates), with known experimental 3D structures, of the studied targets (Fgb1 and Fophy), similarity searches against PDB were performed using the BLAST server (free web service, NCBI, Bethesda, MD, USA) [29]. The Clustal Omega program server (free web service, EMBL-EBI, Hinxton, UK) [30] was used to align the proteins’ sequences of the best selected templates and the studied targets. For each target protein, a homology model was generated by the automated protein structure homology-modeling server SWISS-MODEL (free web service, SIB, Lausanne, Switzerland) [31]. The Ramachandran plot generated by PROCHECK [32] via PDBsum database (free web service, EMBL-EBI, Hinxton, UK) [33], and local quality and comparison plots via SWISS-MODEL, were utilized to check the validity of the built models. DS (version 16 (academic version), FSO, Oujda, Morocco) was used to visualize the three-dimensional structure and to predict the active site of the resultant models.

#### 3.4.3. Molecular Docking

Molecular docking studies of the most and the least active antifungal compounds against the homology models of the Fgb1 and Fophy proteins were performed using the SwissDock server (free web service, SIB, Lausanne, Switzerland) [34]. The CHARMM force field was used in the calculations, keeping the default parameter settings. The analysis of docking results was carried out using the UCSF Chimera molecular viewer (version 1.12, FSO, Oujda, Morocco) [35].

## 4. Conclusions

A series of twelve Schiff base derivatives were synthesized and characterized by FT-IR, mass spectroscopy, ^1^H- and ^13^C-NMR. The preliminary antibacterial and antifungal screening against *E. coli* bacterium and *Fusarium oxysporum f.sp albedinis (F.o.a)* fungus, respectively, proved that most of these compounds exhibited good to excellent antifungal activity, but very weak antibacterial effect. Furthermore, theoretical investigations by homology modeling and docking studies were undertaken in order to illustrate and understand the possible binding mode of the most and least active compounds towards the Fgb1 and Fophy proteins. The results obtained were found to be in good agreement with the experimental findings in both cases. However, more appropriate in vitro experiments on the inhibition of Fgb1 or Fophy with the novel Schiff base compounds should be carried out, and their interactions should be confirmed by others, performing methods such as free-energy calculations and molecular dynamic simulations.

## Figures and Tables

**Table 1:** Rate of inhibition of the growth of *F.o.a* according to the concentration of the tested compounds (0.01–1.50 µg·mL^−1^) and the MIC values.

Comp.	Rate of Inhibition (%)									MIC (µg·mL^−1^)
	0.01	0.05	0.10	0.15	0.25	0.50	0.75	1.00	1.50	
**1**	0	0	7	26	55	78	86	100	100	0.10
**2**	0	0	0	0	0	0	0	15	27	0.90
**3**	8	22	53	82	100	100	100	100	100	**0.02**
**4**	0	0	0	0	7	7	7	12	15	0.25
**5**	0	0	0	0	0	10	9	9	15	0.30
**6**	0	13	13	17	23	46	72	100	100	**0.04**
**7**	0	10	33	49	55	67	89	100	100	**0.04**
**8**	0	0	0	9	12	14	14	23	37	0.12
**9**	0	0	0	0	7	7	8	13	15	0.25
**10**	0	0	0	0	12	15	14	17	23	0.20
**11**	0	0	15	33	47	62	66	79	90	0.08
**12**	0	12	12	24	26	48	61	69	82	**0.04**
**(-)**	-	-	-	-	-	-	-	-	-	**-**

(-): Negative control = DMSO/EtOH + distilled water.

**Table 2:** Docking results against the homology model of the Fophy protein.

Compound	ΔG_binding_ (kcal/mol)	Interaction	Bond Length (Å)
**2**	−5.84	N(pyridine)–(H)Ser431	2.33
**3**	−6.78	N(pyridine)–(H)Ser431	2.15
		O(NO_2_)–(O)Glu422	3.16
		O(NO_2_)–(O)Asp329	3.41
		O(NO_2_)–(HN)Ile326	3.54
